# Evaluation of nuclear abnormalities in buccal mucosa cells of grazing cattle from pastures routinely treated with 2,4-D: an exploratory field study

**DOI:** 10.1007/s10661-026-15851-8

**Published:** 2026-08-24

**Authors:** Cimara Sales Vieira, Rafael Carneiro Silva, Juliana Ferreira da Silva, Iasmim Adelardo Queiroz, Andreia dos Santos Moraes, Damiana Mírian da Cruz e Cunha, Aparecido Divino da Cruz, Alex Silva da Cruz

**Affiliations:** 1 Núcleo de Pesquisas Replicon, Programa de Pós-graduação em Genética, Pontifícia Universidade Católica de Goiás, Escola de Ciências Médicas e da Vida, Goiânia, GO Brazil; 2 Programa de Pós-graduação em Genética, Pontifícia Universidade Católica de Goiás, Escola de Ciências Médicas e da Vida, Goiânia, GO Brazil

**Keywords:** Buccal micronucleus assay, Nuclear abnormalities, Sentinel livestock, Environmental biomonitoring, One Health, Physiological indicators

## Abstract

The herbicide 2,4-dichlorophenoxyacetic acid (2,4-D) is widely used in agricultural systems, raising concerns about its potential genotoxic effects on non-target organisms. This cross-sectional observational field study evaluated nuclear abnormalities in exfoliated buccal mucosa cells from 36 grazing cattle maintained on a farm where 2,4-D was routinely used in Goiás, Brazil, and investigated their relationship with physiological variables, including rectal temperature, body weight, and body condition score (BCS). A total of 1,000 cells per animal (36,000 cells) were analyzed to quantify micronuclei and other nuclear abnormalities. Statistical analyses included the Shapiro–Wilk test, Mann–Whitney test, rank-biserial correlation, Spearman correlation, Benjamini–Hochberg correction for multiple comparisons, and multivariable Poisson regression with robust standard errors. Overall, low frequencies of micronuclei and other nuclear abnormalities were observed. Although nominal associations were identified in the unadjusted analyses, none remained statistically significant after Benjamini–Hochberg correction for multiple comparisons. In the multivariable Poisson regression model, body condition score remained inversely associated with micronucleus frequency (incidence rate ratio = 0.274; 95% confidence interval: 0.128–0.587; *p* = < 0.001). These findings suggest that physiological status may contribute to variation in cytological biomarkers under field conditions. Because individual exposure to 2,4-D was not directly measured and no untreated control group was included, the findings should be interpreted as exploratory. This study contributes to the use of cattle as sentinel organisms in environmental biomonitoring and reinforces the need for future studies incorporating direct exposure assessment and complementary biomarkers within the One Health framework.

## Introduction

The intensive use of pesticides in modern agriculture has raised global concerns regarding their effects on environmental, animal, and human health. Among these compounds, 2,4-dichlorophenoxyacetic acid (2,4-D) is one of the most widely used herbicides for post-emergence control of broadleaf weeds due to its effectiveness, relatively low cost, and broad applicability in agricultural systems (Peterson et al., 2016). However, the widespread use of 2,4-D increases the potential for environmental dispersion beyond target areas, potentially affecting non-target organisms and contributing to contamination of soil, water, and biological systems (Carvalho, 2017; EPA, 2023). Furthermore, the International Agency for Research on Cancer (IARC) has classified 2,4-D as "possibly carcinogenic to humans" (Group 2B), reinforcing the importance of investigating biological responses in agricultural settings where the herbicide is routinely applied (IARC, 2017).

Environmental biomonitoring using sentinel organisms represents an important strategy for detecting biological responses associated with environmental contamination in agricultural ecosystems. Due to their permanent contact with pasture, water sources, and the surrounding agricultural environment, cattle may serve as suitable sentinel organisms for monitoring the biological consequences of agricultural practices. In addition, the collection of exfoliated buccal mucosa cells is a non-invasive, practical, and low-cost approach that has been widely employed in environmental biomonitoring and genotoxicity studies (Ferré et al., 2024; Holland et al., 2008).

The micronucleus assay is recognized as a sensitive and reliable tool for detecting chromosomal damage, genomic instability, and cellular responses associated with exposure to physical, chemical, and biological stressors (Fenech, 2000). In exfoliated epithelial cells, this method enables the identification of nuclear abnormalities, including micronuclei, binucleated cells, karyorrhexis, karyolysis, and broken-egg formations, which reflect different biological processes related to chromosomal damage, mitotic disturbances, and cell death (Holland et al., 2008; Meneses de Araújo et al., 2018). These nuclear abnormalities have been widely investigated as biomarkers in environmental and occupational studies involving pesticides and other potentially genotoxic agents.

In livestock systems, physiological conditions may influence cellular responses to environmental stressors. Factors such as body condition, nutritional status, metabolic balance, and thermal stress can affect oxidative metabolism and cellular homeostasis, potentially influencing the occurrence of nuclear abnormalities (Almeida et al., 2020; Idris et al., 2021). Therefore, the integrated evaluation of nuclear abnormalities and physiological indicators may provide a broader understanding of biological responses observed under field conditions in agricultural environments.

This study investigated the occurrence of nuclear abnormalities in buccal mucosa cells of cattle raised on pastures routinely treated with 2,4-D. Additionally, the relationships between nuclear abnormalities and physiological indicators were evaluated using non-parametric analyses, correlation tests, and multivariate statistical models. The findings provide information that may contribute to environmental biomonitoring and support future investigations within the One Health framework by integrating animal health, agricultural practices, and environmental quality.

## Materials and methods

**Fig. 1:**
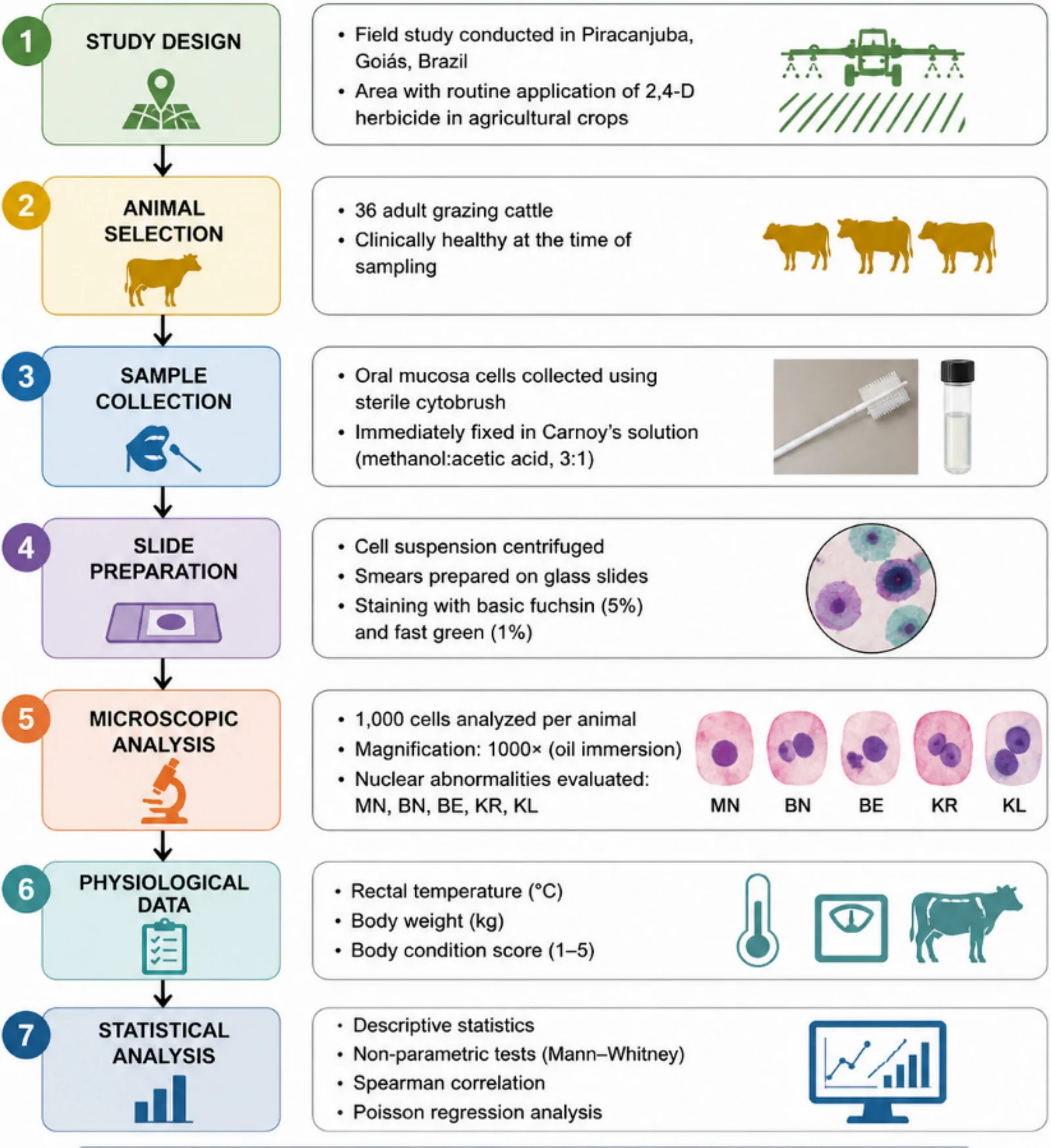
Workflow of the experimental design, sample collection, laboratory procedures, and statistical analyses performed in cattle maintained on a farm where 2,4-D was routinely used

The overall experimental design and analytical workflow of this study are summarized in Fig. 1.

### Study area and animals

This cross-sectional observational field study was designed to evaluate nuclear abnormalities in buccal mucosa cells of cattle maintained under routine agricultural conditions on a farm where the herbicide 2,4-D was regularly used for broadleaf weed control. No experimental herbicide application or controlled exposure was performed as part of the study, and individual exposure to 2,4-D or other environmental contaminants was not directly measured.

A cross-sectional observational study was conducted on a dairy cattle farm located in Piracanjuba, Goiás, Brazil. The farm routinely applied the herbicide 2,4-D for broadleaf weed control during the 2023 production cycle. The study included all 36 adult female Girolando cows present on the farm at the time of sampling, aged between 2 and 5 years. Therefore, no inclusion or exclusion criteria were applied beyond animal availability during routine farm management.

At the time of sampling, body weight, rectal temperature, and body condition score (BCS) were recorded for each animal. A routine clinical evaluation was also performed, including assessment of fecal consistency and hydration status. Most animals presented normal fecal consistency and adequate hydration, with only isolated observations of soft feces or mild dehydration. As part of the farm's routine dairy management, all animals received oxytocin before milking. This management practice was unrelated to the objectives of the present study and was not considered an experimental intervention.

Detailed farm records regarding the commercial formulation, application rate, application dates, application frequency, treated area, grazing interval, rainfall, and other environmental conditions were not available for this study. Information regarding reproductive status, duration of residence on the farm, and water source was not available.

The investigation aimed to evaluate nuclear abnormalities in cattle maintained under field conditions in an agricultural environment where 2,4-D was routinely used. Individual exposure to 2,4-D or other environmental contaminants was not directly measured, and no experimental herbicide application or treatment was performed for the purposes of this study. All procedures were approved by the Institutional Animal Ethics Committee (CEUA Protocol No. 8705240423).

### Sample collection, slide preparation and staining

Exfoliated buccal mucosa cells were collected using sterile cytology brushes. Samples were obtained from the upper inner cheek by gentle rotational scraping and immediately transferred to Carnoy's fixative (methanol:acetic acid, 3:1, v/v). The samples were transported to the laboratory and stored under appropriate conditions until processing.

Cell suspensions were subsequently centrifuged, washed, and fixed according to standardized laboratory procedures. Smears were prepared on clean glass slides, air-dried, and stained with basic fuchsin and fast green following established protocols for the evaluation of nuclear abnormalities in exfoliated epithelial cells.

### Micronucleus and nuclear abnormality assessment

**Fig. 2:**
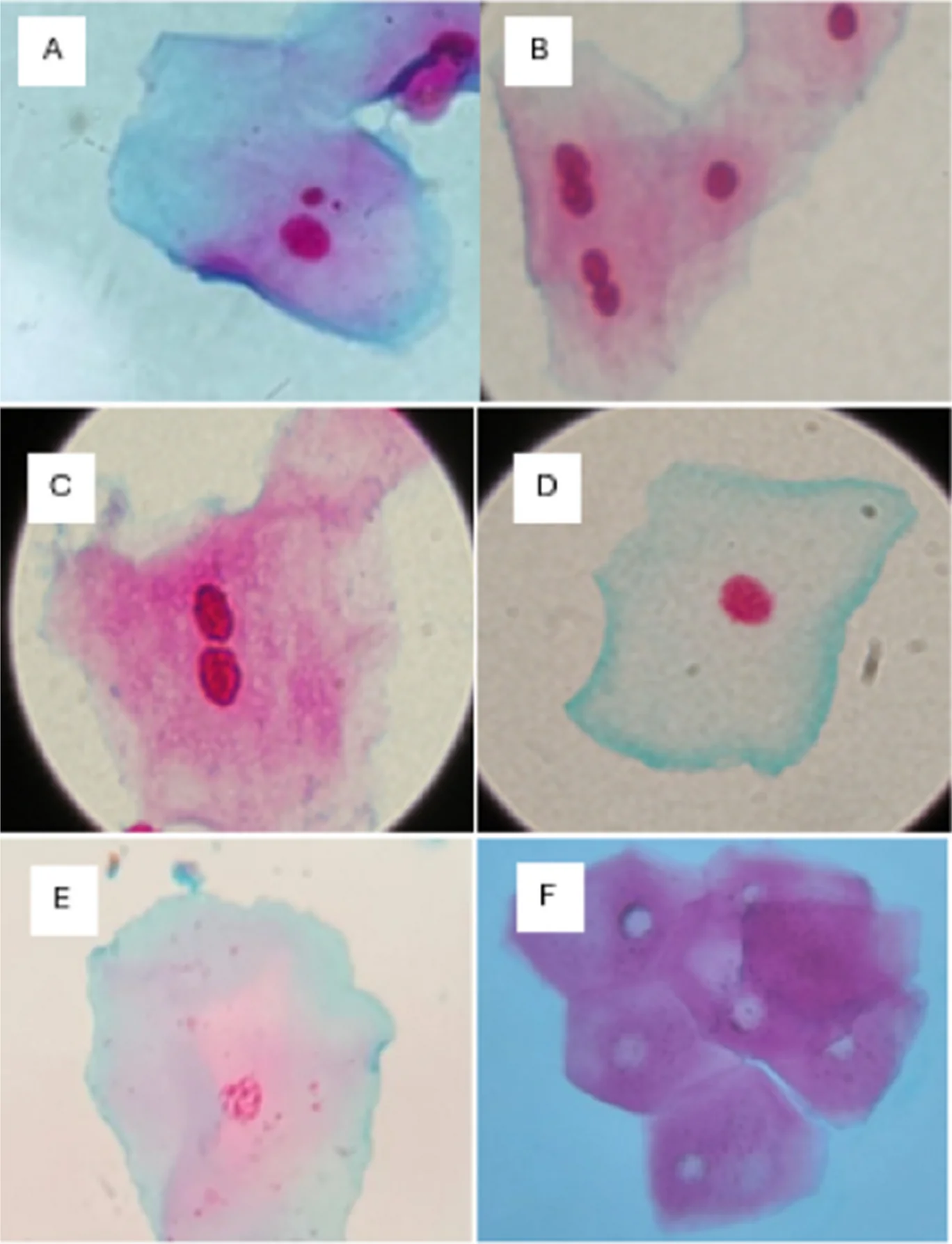
Representative nuclear abnormalities observed in exfoliated buccal mucosa cells of cattle stained with basic fuchsin and fast green. **A** Micronucleus (MN); **B** Broken-egg formation (BE); **C** Binucleated cell (BN); **D** Karyorrhexis (KR); **E** Karyolysis (KL); **F** Normal epithelial cell

For each animal, 1,000 exfoliated buccal epithelial cells were examined under light microscopy at 1000 × magnification, totaling 36,000 analyzed cells. Nuclear abnormalities were identified according to established morphological criteria and included micronuclei (MN), binucleated cells (BN), broken-egg formations (BE), karyorrhexis (KR), and karyolysis (KL) (Fig. 2).

The frequencies of each nuclear abnormality were recorded separately for subsequent statistical analyses.

### Physiological indicators

Rectal temperature, body weight, and body condition score (BCS) were recorded for each animal at the time of sample collection. Rectal temperature was measured using a calibrated digital veterinary thermometer. Body weight was estimated using a bovine weight tape positioned around the thoracic girth according to the manufacturer's instructions. All measurements were obtained by the same trained evaluator on the same day under routine farm management conditions.

Body condition score was assessed by visual inspection and palpation of anatomical landmarks, including the lumbar region, ribs, hooks, pins, tail head, and neck base, using the conventional 1-to-5 scoring system, in which higher scores indicate greater body fat reserves. BCS evaluation was performed by the same trained evaluator to minimize observer variability. These physiological variables were included to investigate their association with the frequencies of nuclear abnormalities under field conditions.

### Statistical analysis

Data were initially recorded on standardized collection forms and subsequently entered into electronic spreadsheets for quality control and organization prior to statistical analysis. Nuclear abnormalities (micronuclei, broken-egg formations, binucleated cells, karyorrhexis, and karyolysis) were recorded separately for each animal.

Descriptive statistics were calculated for physiological variables and nuclear abnormalities, including mean, standard deviation, median, minimum and maximum values, and 95% confidence intervals. Data normality was assessed using the Shapiro–Wilk test.

Because most variables did not follow a normal distribution, comparisons between physiological groups were performed using the Mann–Whitney U test, and effect sizes were estimated using the rank-biserial correlation coefficient (r < sub > rb </sub >). To account for multiple comparisons, *p*-values were adjusted using the Benjamini–Hochberg false discovery rate (FDR) procedure.

Associations between physiological variables and nuclear abnormalities were evaluated using Spearman's rank correlation coefficient.

Generalized linear models with a Poisson distribution and log link function were fitted to evaluate the associations between physiological variables and the frequencies of nuclear abnormalities. The logarithm of the total number of analyzed cells was included as an offset. Rectal temperature, body weight, and body condition score (BCS) were included as explanatory variables. Model dispersion was assessed, and heteroscedasticity-consistent (robust) standard errors were used to account for potential mild overdispersion. Results are presented as incidence rate ratios (IRRs) with 95% confidence intervals (95% CIs).

All statistical analyses were performed using R version 4.5.0 (R Foundation for Statistical Computing, Vienna, Austria). Statistical significance was set at *p* < 0.05.

## Results

A total of 36 adult female Girolando cattle were evaluated, with 1,000 exfoliated buccal epithelial cells analyzed per animal, totaling 36,000 cells. Descriptive statistics are presented in Table 1. Karyolysis and karyorrhexis were the most frequent nuclear abnormalities, whereas micronuclei and broken-egg formations occurred at comparatively lower frequencies.

**Table 1:** Descriptive statistics of nuclear abnormalities in oral mucosa cells of cattle (*n* = 36)

Variable	Mean ± SD	Median	Min–Max	95% CI
Micronuclei (MN)	1.28 ± 1.36	1.00	0–5	0.82–1.74
Broken-egg (BE)	0.19 ± 0.47	0.00	0–2	0.03–0.35
Binucleated cells (BN)	2.47 ± 2.01	2.00	0–8	1.79–3.15
Karyorrhexis (KR)	5.83 ± 4.29	5.00	0–18	4.38–7.28
Karyolysis (KL)	13.33 ± 7.03	12.00	2–30	10.97–15.69

*MN*,  micronuclei; *BE*,  broken-egg formations; *BN*, binucleated cells; *KR*,  karyorrhexis; *KL*, karyolysis; *SD*, standard deviation; *CI*,  confidence interval

Comparisons of nuclear abnormalities according to physiological variables are presented in Table 2. In the unadjusted analyses, cattle with rectal temperatures ≥ 38.0 °C showed higher frequencies of binucleated cells than those with temperatures ≤ 37.9 °C (*p* = 0.025), whereas animals with body condition scores (BCS) ≥ 3.5 showed lower frequencies of karyorrhexis than those with BCS ≤ 3.0 (*p* = 0.022). Body weight also showed a tendency toward association with broken-egg formations (*p* = 0.078). However, after adjustment for multiple comparisons using the Benjamini–Hochberg false discovery rate procedure, none of these associations remained statistically significant.

**Table 2:** Comparison of nuclear abnormalities according to physiological variables

Variable	Group	Mean ± SD	*p*-value	Adjusted *p*-value (FDR)	Rank-biserial correlation
MN	≤ 37.9 °C	1.29 ± 1.64	0.636	0.883	0.094
	≥ 38.0 °C	1.27 ± 1.20			
BE	≤ 37.9 °C	0.14 ± 0.36	0.745	0.883	0.045
	≥ 38.0 °C	0.23 ± 0.53			
BN	≤ 37.9 °C	1.57 ± 1.83	0.025	0.190	0.445
	≥ 38.0 °C	3.05 ± 1.94			
KR	BCS ≤ 3.0	8.89 ± 4.20	0.022	0.190	− 0.519
	BCS ≥ 3.5	4.81 ± 3.88			

*MN*,  micronuclei; *BE*,  broken-egg formations; *BN*,  binucleated cells; *KR*,  karyorrhexis; *BCS*,  body condition score. *P*-values were adjusted using the Benjamini–Hochberg false discovery rate (FDR**)** procedure. Rank-biserial correlation is reported as the effect size for the Mann–Whitney test. None of the comparisons remained statistically significant after adjustment for multiple comparisons

Spearman correlation analyses are presented in Table 3. In the unadjusted analyses, body weight was positively correlated with broken-egg formations (*ρ* = 0.352, *p* = 0.573) and negatively correlated with karyorrhexis (*ρ* =  − 0.419, *p* = 0.035). Likewise, body condition score was negatively correlated with karyorrhexis (*ρ* =  − 0.423, *p* = 0.011) and micronucleus frequency (*ρ* =  − 0.337, *p* = 0.010). After adjustment for multiple testing using the Benjamini–Hochberg procedure, none of these correlations remained statistically significant.

**Table 3:** Spearman correlations between physiological variables and nuclear abnormalities

Variable Pair	Spearman (ρ)	*p*-value	Adjusted *p*-value (FDR)
Temperature × MN	0.079	0.648	0.748
Temperature × BE	0.097	0.20	0.748
Weight × BE	0.352	0.573	0.166
Weight × KR	− 0.419	0.035	0.082
BCS × KR	− 0.423	0.011	0.082
BCS × MN	− 0.337	0.010	0.166

*MN*, micronuclei; *BE*, broken-egg formations; *KR*, karyorrhexis; *BCS*, body condition score; *ρ*, Spearman correlation coefficient. *P*-values were adjusted using the Benjamini–Hochberg false discovery rate (FDR) procedure. None of the correlations remained statistically significant after correction for multiple comparisons

To further evaluate the independent associations between physiological variables and micronucleus frequency, a multivariable Poisson regression model with robust (HC0) standard errors was fitted (Table 4). In the adjusted model, body condition score remained independently associated with micronucleus frequency, with higher BCS values associated with lower micronucleus counts (IRR = 0.274; 95% CI: 0.128–0.587; *p* < 0.001). Rectal temperature (IRR = 1.009; 95% CI: 0.512–1.986; *p* = 0.980) and body weight (IRR = 1.001; 95% CI: 0.996–1.005; *p* = 0.732) were not significantly associated with micronucleus frequency in the adjusted model.

**Table 4:** Poisson regression analysis for predictors of micronucleus frequency

Variable	IRR	95% CI	*p*-value
Rectal Temperature	1.009	0.512–1.986	0.980
Weight	1.001	0.996–1.005	0.732
BCS	0.274	0.128–0.587	< 0.001

*IRR*, incidence rate ratio; *CI*, confidence interval; *BCS*, body condition score. Estimates were obtained from a multivariable Poisson regression model with robust (HC0) standard errors

## Discussion

This study evaluated nuclear abnormalities in exfoliated buccal epithelial cells from grazing cattle maintained on a farm where 2,4-D was routinely used for weed control and investigated whether physiological variables were associated with these cytological endpoints. Overall, the frequencies of micronuclei and other nuclear abnormalities were low. Although some associations were observed in the unadjusted analyses, these did not remain statistically significant after correction for multiple comparisons using the Benjamini–Hochberg false discovery rate procedure. In contrast, body condition score remained independently associated with micronucleus frequency in the multivariable Poisson regression model, suggesting that physiological status may contribute to variation in cytological responses under field conditions.

The absence of a significant association between rectal temperature and micronucleus frequency suggests that thermal variation alone was not associated with chromosomal damage in this population. Although cattle with rectal temperatures ≥ 38.0 °C showed higher frequencies of binucleated cells in the unadjusted analysis, this association was no longer statistically significant after correction for multiple comparisons. Therefore, this finding should be considered exploratory and interpreted with caution until confirmed by studies with larger sample sizes (Fenech, 2000; Holland et al., 2008).

Body condition score remained inversely associated with micronucleus frequency in the multivariable Poisson regression model. Animals with higher body condition scores presented lower micronucleus frequencies after adjustment for rectal temperature and body weight. Body condition score is commonly considered an indicator of nutritional status, metabolic balance, and overall physiological condition in cattle. Therefore, this association suggests that physiological condition may influence variation in micronucleus frequency. However, given the observational nature of the present study, this finding should not be interpreted as evidence of a causal relationship and requires confirmation in future studies (Ferré et al., 2024).

Negative associations between body weight and karyorrhexis and between body condition score and karyorrhexis were observed in the unadjusted analyses. However, these associations were not retained after correction for multiple comparisons and should therefore be interpreted with caution. Karyorrhexis is commonly regarded as an indicator of nuclear fragmentation during cell death (Fenech, 2000; Holland et al., 2008), but the present results do not provide sufficient evidence to support an association between this endpoint and physiological status under the evaluated conditions.

The low frequency of micronuclei observed in this study may reflect the complexity of field conditions. Unlike controlled experimental models, commercial grazing systems involve heterogeneous environmental conditions, variable management practices, and potential variation in contact with agricultural chemicals. In addition, individual differences in nutritional status, metabolism, and physiology may contribute to biological variability (Almeida et al., 2020; Grossmann, 2009; Peterson et al., 2016). These factors may reduce the ability to detect subtle cytogenetic responses in cross-sectional field studies.

Previous studies have demonstrated that 2,4-D can induce DNA damage in experimental models, including cultured human cells and laboratory animals (IARC, 2017; Peterson et al., 2016). However, these studies generally involve controlled exposure conditions that differ substantially from routine agricultural settings. In the present study, individual exposure to 2,4-D was not directly measured, and no untreated comparison group was included. Therefore, the findings should not be interpreted as evidence supporting or refuting genotoxic effects of 2,4-D but rather as an evaluation of nuclear abnormalities in cattle maintained under routine field conditions on a farm where the herbicide was regularly used.

Environmental and physiological factors may influence biomarkers of genomic damage. Heat stress has been associated with oxidative stress and metabolic alterations in livestock (Almeida et al., 2020; Idris et al., 2021). Although an apparent increase in binucleated cells was observed among animals with higher rectal temperatures in the unadjusted analyses, this association was not maintained after correction for multiple testing. Consequently, additional studies are needed to determine whether physiological stress contributes to variation in cytological endpoints under field conditions.

Importantly, the absence of statistically significant associations after correction for multiple comparisons should not be interpreted as definitive evidence for the absence of biological effects. Rather, these findings indicate that, under the evaluated field conditions and within the statistical power of the present study, no robust associations were identified for most of the evaluated biomarkers.

Although the associations involving binucleated cells, karyorrhexis, body weight, and body condition score were not retained after correction for multiple comparisons, these observations should not be dismissed. Instead, they may represent biological patterns that could not be detected with sufficient statistical confidence in the present sample. Cytogenetic biomarkers are influenced by multiple physiological and environmental factors, and subtle biological variation may become more evident in studies with larger sample sizes, longitudinal follow-up, and direct assessment of environmental exposures. Therefore, these findings should be regarded as hypothesis-generating rather than conclusive evidence of biological effects.

The main limitations of this study include its cross-sectional design, the relatively small sample size, the absence of an untreated control group, and the lack of direct measurements of individual exposure to 2,4-D or other environmental contaminants. In addition, pesticide residues were not quantified in environmental or biological samples, limiting interpretation of exposure-response relationships. Future studies should include longitudinal sampling, larger populations, untreated comparison groups whenever feasible, direct exposure assessment, and complementary biomarkers such as oxidative stress indicators and DNA damage assays (Araldi et al., 2015; Fenech, 2000).

Overall, the present findings indicate that nuclear abnormalities occurred at low frequencies in cattle maintained under routine field conditions. Although most associations identified in the unadjusted analyses were not maintained after correction for multiple comparisons, the independent association between body condition score and micronucleus frequency observed in the multivariable model suggests that physiological status may contribute to variation in cytological biomarkers. These findings support the inclusion of physiological variables in future environmental biomonitoring studies using livestock as sentinel organisms and warrant confirmation in larger studies incorporating direct exposure assessment and additional biomarkers.

## Conclusion

This study evaluated nuclear abnormalities in buccal epithelial cells from grazing cattle maintained on a farm where 2,4-D was routinely used for weed control. Overall, low frequencies of micronuclei and other nuclear abnormalities were observed under the evaluated field conditions, and no robust associations were identified between the evaluated physiological variables and cytological biomarkers after correction for multiple comparisons. However, body condition score remained independently associated with micronucleus frequency in the multivariable Poisson regression model, suggesting that physiological status may contribute to variation in cytological responses under field conditions.

These findings highlight the importance of considering physiological variables when interpreting cytogenetic biomarkers in environmental biomonitoring studies involving livestock. Because this was a cross-sectional observational study without direct measurement of individual pesticide exposure or an untreated control group, the results should be considered exploratory and should not be interpreted as evidence for or against genotoxic effects of 2,4-D. Future studies incorporating larger sample sizes, longitudinal designs, direct exposure assessment, and complementary biomarkers will be important to further investigate the relationships between agricultural environments, physiological condition, and cytogenetic responses in cattle.

## Data Availability

The datasets generated and analyzed during the current study are available from the corresponding author on reasonable request.
